# Decoupling Behavioral Domains via Kynurenic Acid Analog Optimization: Implications for Schizophrenia and Parkinson’s Disease Therapeutics

**DOI:** 10.3390/cells14130973

**Published:** 2025-06-25

**Authors:** Diána Martos, Bálint Lőrinczi, István Szatmári, László Vécsei, Masaru Tanaka

**Affiliations:** 1 HUN-REN-SZTE Neuroscience Research Group, Hungarian Research Network, University of Szeged Danube Neuroscience Research Laboratory, Tisza Lajos krt. 113, H-6725 Szeged, Hungary; martos.diana@med.u-szeged.hu; 2 Institute of Pharmaceutical Chemistry and HUN-REN-SZTE Stereochemistry Research Group, University of Szeged, Eötvös u. 6, H-6720 Szeged, Hungary; lorinczi.balint@szte.hu (B.L.); szatmari.istvan@szte.hu (I.S.); 3 Department of Neurology, Albert Szent-Györgyi Medical School, University of Szeged, Semmelweis u. 6, H-6725 Szeged, Hungary

**Keywords:** kynurenic acid, neuroprotective agents, drug design, structure–activity relationship, motor activity, glutamatergic system, animal models, schizophrenia, Parkinson’s disease

## Abstract

Kynurenic acid (KYNA), a putative neuroprotective agent, modulates glutamatergic pathways in schizophrenia and Parkinson’s disease but is limited by acute motor activity impairments (e.g., ataxia). Research leveraging animal disease models explores its structure–activity relationship to enhance therapeutic efficacy while mitigating adverse effects, addressing global neuropsychiatric disorders affecting over 1 billion people. Structural analogs of KYNA (SZR-72, SZR-73, and SZR-81) were designed to uncouple therapeutic benefits from motor toxicity; yet, systematic comparisons of their acute behavioral profiles remain unexplored. Here, we assess the motor safety, time-dependent effects, and therapeutic potential of these analogs in mice. Using acute intracerebroventricular dosing, we evaluated motor coordination (rotarod), locomotor activity (open-field), and stereotypic behaviors. KYNA induced significant ataxia and stereotypic behaviors at 15 min, resolving by 45 min. In contrast, all analogs avoided acute motor deficits, with SZR-73 maintaining baseline rotarod performance and eliciting a delayed decrease in ambulation and inquisitiveness in open-field assays. These findings demonstrate that the structural optimization of KYNA successfully mitigates motor toxicity while retaining neuromodulatory activity. Here, we show that SZR-73 emerges as a lead candidate, combining transient therapeutic effects with preserved motor coordination. This study advances the development of safer neuroactive compounds, bridging a critical gap between preclinical innovation and clinical translation. Future work must validate chronic efficacy, disease relevance, and mechanistic targets to harness the full potential of KYNA analogs in treating complex neuropsychiatric disorders.

## 1. Introduction

Neurological and psychiatric disorders, including Alzheimer’s disease (AD), Parkinson’s disease (PD), schizophrenia (SCZ), and major depressive disorder, represent a profound and growing global health crisis [1,2,3,4,5,6]. Over 1 billion people worldwide are affected by these conditions, which collectively account for nearly 15% of the global disease burden [1,7,8,9,10]. AD alone, the leading cause of dementia, is projected to afflict 152 million individuals by 2050, while psychiatric disorders such as SCZ and depression contribute significantly to disability-adjusted life years, reducing productivity and quality of life [3,11,12,13,14,15,16,17,18,19]. This study addresses these gaps by exploring kynurenic acid (KYNA) analogs, which modulate neuroinflammatory and glutamatergic pathways, as a novel strategy to balance efficacy and safety in a critical step toward alleviating the socioeconomic and humanistic toll of these debilitating disorders [20,21,22,23,24].

The kynurenine (KYN) pathway, a critical metabolic route for tryptophan degradation, plays a dual role in shaping brain health by modulating neuroinflammatory and neuroprotective processes [25,26,27,28,29]. Central to this balance is KYNA, a neuroactive metabolite that exerts multifaceted effects on neuronal and immune function. KYNA acts as an endogenous antagonist of the N-methyl-D-aspartate (NMDA) receptor, dampening glutamate excitotoxicity, a key driver of neurodegeneration in conditions like AD and stroke [30,31,32,33,34]. By regulating glutamatergic signaling, KYNA prevents excessive calcium influx and neuronal apoptosis, offering protection against oxidative stress, which is further mitigated through its scavenging of free radicals and enhancement of antioxidant defenses [30,32,35,36,37,38]. Concurrently, KYNA modulates neuroimmune interactions, suppressing pro-inflammatory cytokine release (e.g., TNF-α, IL-6) and microglial activation, thereby attenuating chronic neuroinflammation linked to disorders such as multiple sclerosis and depression [27,39,40,41,42,43]. However, the pathway’s duality is underscored by its context-dependent outcomes: while KYNA promotes neuroprotection, imbalances in KYN metabolites (e.g., elevated quinolinic acid) can exacerbate neurodegeneration [26,40,44,45,46]. This intricate interplay positions KYNA as a pivotal mediator at the intersection of inflammation and resilience, making the KYN pathway a promising therapeutic target [27,39,47,48]. Strategies to enhance KYNA production or deliver its analogs aim to recalibrate this equilibrium, addressing both the inflammatory cascades and oxidative damage underlying neurological and psychiatric diseases [27,47,49]. Thus, understanding KYNA’s dual role not only clarifies pathogenic mechanisms, but also illuminates novel avenues for interventions that harmonize neuroprotection with anti-inflammatory efficacy [39,50,51].

**Figure 1:**
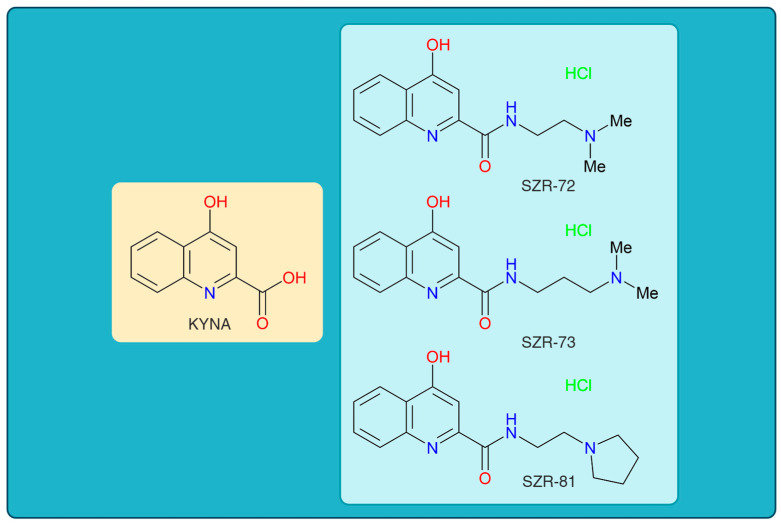
The chemical structures of kynurenic acid and its analogs. KYNA, kynurenic acid; SZR-72, *N*-(2-(dimethylamino)ethyl)-4-hydroxyquinoline-2-carboxamide hydrochloride; SZR-73, *N*-(3-(dimethylamino)propyl)-4-hydroxyquinoline-2-carboxamide hydrochloride; SZR-81, 4-hydroxy-*N*-(2-(pyrrolidin-1-yl)ethyl)quinoline-2-carboxamide hydrochloride.

KYNA has garnered interest in its neuroprotective and neuromodulatory properties, particularly in neurological and psychiatric disorders such as SCZ, epilepsy, and neurodegeneration [49,52,53]. However, its clinical translation is hampered by acute motor side effects, including ataxia, stereotypic behaviors, and sedation, which emerge within minutes of administration [54,55,56]. These transient yet dose-limiting impairments, observed in preclinical models, risk overshadowing KYNA’s therapeutic benefits, as motor dysfunction could compromise patient safety and adherence [36,57,58]. To address this, structural modifications of KYNA led to novel analogs—SZR-72, SZR-73, and SZR-81—designed to decouple therapeutic benefits from adverse outcomes (Figure 1) [59,60]. Rational design focuses on altering KYNA’s core structure, such as side chain additions or functional group substitutions, to enhance receptor selectivity, optimize BBB permeability, or reduce interactions with motor circuitry [61,62,63].

This study aims to address these gaps through three objectives. First, to rigorously assess motor safety by quantifying acute side effects (e.g., ataxia, stereotype) across analogs using standardized tests like rotarod and open-field assays. Second, to map time-dependent behavioral profiles by analyzing outcomes at 15 and 45 min post-injection intervals, revealing whether the effects are transient or sustained. Third, to evaluate the therapeutic potential of analogs by contrasting their motor tolerability with KYNA’s known neuroprotective properties. By integrating dose–response analyses and time–course experiments, this study systematically explores how structural tweaks—such as side chain additions in SZR-73—alter pharmacodynamics to mitigate motor impairments while retaining beneficial activity. This research not only clarifies the translational promise of KYNA analogs, but also establishes a framework for optimizing neuromodulators through structure-guided innovation.

## 2. Materials and Methods

### 2.1. Study Design and Ethical Framework

This study used a controlled acute dosing paradigm to examine the motor safety and behavioral effects of KYNA and its analogs in 10–12-week-old (25–30 g) male C57BL/6 J mice (n = 7–10 mice/group). All procedures were approved by the Institutional Animal Care and Use Committee (IACUC) and the Ethical Committee for the Protection of Animals in Research of the University of Szeged (approval code: XI/352/2012, approval date: 1 March 2012), in accordance with the Regulations of the Faculty of Medicine, University of Szeged; the Hungarian Health Committee; Government Decree 40/2013 (II.14.), with most sections effective from 26 March 2014 and 2 September 2017; and the European Communities Council Directive 2010/63/EU of 22 September 2010. Mice received acute intracerebroventricular injections (i.c.v.) of KYNA or equimolar analogs, with behavioral evaluations at 15 and 45 min post injection [64,65,66,67]. Motor coordination was measured via the rotarod, and open-field assays assessed locomotion and exploration. Stereotype and ataxia were rated by blind observers. No severe adverse events were noted, supporting translational relevance for acute neuropharmacological screening.

### 2.2. Animal Models and Treatment Protocols

Male C57BL/6J mice (Mus musculus), sourced from Charles River Laboratories (Erkrath, Germany), aged 10–12 weeks and weighing between 25 and 30 g, were employed in the study. Animals were group-housed, with no more than five individuals per standard cage. They were maintained under controlled environmental conditions: a 12 h light/dark cycle, ambient temperature of 24 ± 1 °C, and relative humidity of 50 ± 10%. Mice had unrestricted access to standard rodent chow and tap water throughout the experimental period. Mice were randomly assigned to five groups: vehicle control 0.9% saline, KYNA (Sigma-Aldrich Ltd., Budapest, Hungary) (0.2 µmol/4 µL), and three equimolar analog groups *N*-(2-(dimethylamino)ethyl)-4-hydroxyquinoline-2-carboxamide hydrochloride (SZR-72), *N*-(3-(dimethylamino)propyl)-4-hydroxyquinoline-2-carboxamide hydrochloride (SZR-73), and 4-hydroxy-*N*-(2-(pyrrolidin-1-yl)ethyl)quinoline-2-carboxamide hydrochloride (SZR-81). Fresh solutions of KYNA and its analogs were prepared by dissolving them in 0.9% aqueous saline and adjusting the pH to 7.4. The analogs were synthesized at the Faculty of Pharmacy, Institute of Pharmaceutical Chemistry, University of Szeged. The i.c.v. injections were performed under 4% Chloral hydrate (Sigma-Aldrich Ltd., Budapest, Hungary) at a dose of 0.4 g/kg body weight anesthesia. A polyethylene cannula (Fisher Scientific, In tramedic Clay Adams polyethylene tube, Budapest, Hungary) was inserted into the right lateral brain ventricle and fixed to the skull using cyanoacrylate (Ferrobond, Budapest, Hungary). Using a stereotaxic apparatus, the stereotaxic coordinates were set at anterior–posterior 0.2 mm and medial–lateral 1.09 mm to the bregma, with the cannula extending 2.3 mm deep under the skull surface. Postoperative analgesia (Rimadyl, 1 mg/kg) was administered to minimize discomfort. A total volume of 4 µL of KYNA, its synthetic derivatives, or sterile saline was administered into the right lateral ventricle of the mouse brain via intracerebroventricular (i.c.v.) infusion. The delivery was performed using a precision infusion pump (KD Scientific, Holliston, MA, USA) at a constant flow rate of 8 µL/min. To verify accurate cannula placement post-experimentally, a 1% methylene blue dye solution was injected through the i.c.v. route [68]. Behavioral testing commenced at 15 and 45 min intervals post injection to assess acute effects. To mitigate bias, group assignments and injections were coded, and experimenters were blinded during data collection. Humane endpoints included immediate euthanasia for severe distress, though no such cases occurred [69,70,71,72,73]. This design prioritized translational relevance by mimicking acute clinical dosing scenarios while adhering to rigorous ethical and procedural standards, ensuring reproducibility in evaluating motor safety and therapeutic potential across KYNA and its structural analogs [69,70,71,73,74].

### 2.3. Behavioral Testing Paradigms

Motor and behavioral outcomes were evaluated using standardized and validated protocols to ensure reliability and reproducibility. Ataxia and stereotype were scored on a standardized 5-point scale (Table 1) by blinded observers to minimize bias [75,76]. Locomotor activity and exploratory behavior were quantified using the open-field test, with automated tracking software analyzing parameters such as total distance traveled, rearing frequency. Additionally, motor coordination was assessed through the rotarod test, where mice were trained and tested on an accelerating rod to measure latency to fall, providing insights into balance and endurance. These assessments were conducted at four time points post administration to capture both the immediate and transient effects of the tested compounds, ensuring a comprehensive evaluation of their behavioral impact.

#### 2.3.1. Ataxia and Stereotype Test

Ataxia and stereotype scoring employed a 5-point scale: ataxia was quantified by gait instability, limb splaying, and balance loss (1 = none, 5 = severe), while stereotype (repetitive movements, e.g., head weaving) was scored based on frequency and intensity (1 = absent, 5 = continuous) [75,77,78]. Observations occurred at 3 × 5 min intervals for 15 and 45 min post injection by trained observers [75,77,78].

#### 2.3.2. Open-Field Tests

The open-field test was performed in a square arena measuring 48 × 48 × 40 cm and illuminated with low-intensity light (approximately 50 lux). The apparatus was fitted with an array of infrared sensors to enable accurate behavioral quantification. Each mouse was initially placed at the center of the arena, and spontaneous locomotion (total distance traveled), exploratory behavior, and vertical activity (rearing) were recorded during two consecutive 15 min sessions. Behavioral data were acquired and analyzed using Conducta 1.0 software (Experimetria Ltd., Budapest, Hungary) [79,80,81].

#### 2.3.3. Rotarod Tests

Motor coordination and balance were assessed using the rotarod apparatus (TSE RotaRod System v4.2.5, TSE Systems GmbH, Bad Homburg, Germany). The device consisted of a horizontally oriented rotating rod (3 cm diameter) that revolved around its longitudinal axis. Mice were required to walk forward on the rod to maintain their balance and avoid falling. The rotation speed at which animals lost balance was monitored with an integrated infrared detection system, and latency to fall was recorded automatically. The comparative analysis of fall latency was used to evaluate motor performance across treatment groups. Before the training phase, mice underwent a one-hour habituation period on the stationary apparatus. On the first two days, they received three training sessions (each lasting 5 min, spaced 15 min apart), which included exposure to a non-moving rod for 60 s followed by trials on the rotating rod set at a constant speed of 5 rpm. On the third day, 15, 40, 60, and 120 min after intraventricular (i.c.v.) injection, the latency at which each mouse fell off the rod in accelerated mode (from 5 to 30 rpm, within 5 min) was recorded [82,83,84,85,86]. All tests were performed in a sound-attenuated room to minimize environmental stress [87,88].

### 2.4. Data Acquisition and Statistical Analysis

Behavioral assessments were conducted using automated tracking systems to ensure precise temporal and spatial resolution. Open-field locomotor and exploratory activities—specifically, total ambulation distance and rearing frequency—were monitored via infrared beam arrays and analyzed using Conducta 1.0 software (Experimetria Ltd., Budapest, Hungary). For motor coordination testing, the rotarod performance was recorded using the TSE RotaRod System, with latency to fall captured automatically through an integrated infrared detection system [89,90,91,92,93]. Ataxia and stereotype scores were manually recorded using a standardized 5-point scale. The statistical analysis under the raw datasets was used as a test of Normality with the Kolmogorov–Smirnov (*p* < 0.05) post hoc test, and Homogeneity of variances with the Levene test. Parametric data (e.g., rotarod latencies, score of stereotype 45 min after the i.c.v injection) were analyzed using one-way ANOVA, with LSD post hoc correction for multiple comparisons. Nonparametric datasets (e.g., score of stereotype/ataxia 15 min after the i.c.v. injection and ambulation distance, rearing count in the open-field tests) were evaluated via Kruskal–Wallis tests. All analyses were performed in the IBM SPSS v 2.0 with significance thresholds set at *p* < 0.05. Data are presented as mean ± SEM or median (IQR), with individual animal data points overlaid in graphical outputs. This rigorous tiered approach ensured statistical robustness, aligning with Animal Research: Reporting of In Vivo Experiments (ARRIVE) guidelines for transparent reporting and minimizing type I/II error risks in preclinical neuropharmacological research [94,95].

### 2.5. KYNA Derivatives’ Synthesis

Compounds were synthesized based on previously published methods [61], utilizing the ethyl ester of KYNA and the appropriate amines (*N*,*N*-dimethylethane-1,2-diamine, *N*,*N*-dimethylpropane-1,3-diamine, and 2-(pyrrolidin-1-yl)ethanamine for SZR-72, SZR-73, and SZR-81, respectively). However, the reactions had to be optimized to support the biological tests and were also implemented into up-to-date green methods: the starting ester was used in 10 mmol (2,17 g) and the reaction was carried out under neat conditions using 2 equivalents of amine- and microwave-assisted heating (80 °C). For isolation, the reaction mixture was cooled in an ice bath and 5 mL EtOAc was added. After filtration, the amides were washed with 2 × 5 mL EtOAc. To acquire their HCl salts, the compounds were dissolved in EtOH, HCl/EtOH (23%) was added until a pH of 1–2 was reached, and it was left to stir overnight. The resulting compounds precipitated from the media and were filtrated under vacuo, giving slightly higher yields compared to previous methods (SZR-72: 87%, SZR-73: 86%, SZR-81: 88%).

## 3. Results

### 3.1. Acute Motor Effects of KYNA and Analogs

**Figure 2:**
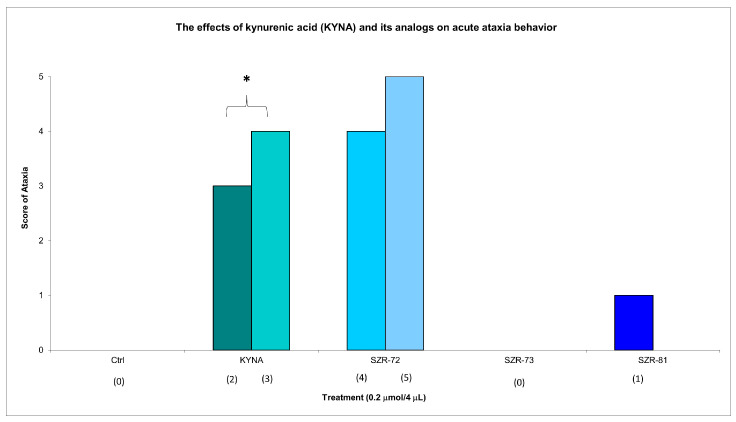
The effects of kynurenic acid and its analogs on acute ataxia behavior. Ataxia symptom scores of KYNA treatment, 0.2 µmol/4 µL KYNA vs. control (Ctrl) group (*p* = 0.014). We show the number of animals and the score categories. The level of significance and the number of animals in groups * *p* < 0.05; N(Ctrl) = 10, N(0.2 µmol/4 µL Kyna) = 10, N(0.2 µmol/4 µL SZR-72) = 10, N(0.2 µmol/4 µL SZR-73) = 10, N(0.2 µmol/4 µL SZR-81) = 10. The statistical analysis was a test of Normality with the Kolmogorov–Smirnov post hoc test, Homogeneity of variances with the Levene test and nonparametrical Kruskal–Wallis test. We show the number of animals in brackets.

KYNA administration (0.2 µmol/4 µL, i.c.v.) elicited significant acute motor impairments at 15 min post injection, with significantly pronounced ataxia (score 3 in two animals, score 4 in three animals). KYNA analog SZR-72 non-significantly induced ataxia (score 4 in one animal and score 5 in one animal); SZR-73 elicit no ataxia; and SZR-81 slightly showed ataxia (score 1 in one animal) at the same dose and timepoint (Figure 2, Table 2). Neither KYNA nor KYNA analogs caused ataxia at 45 min post injection (Table 2).

**Figure 3:**
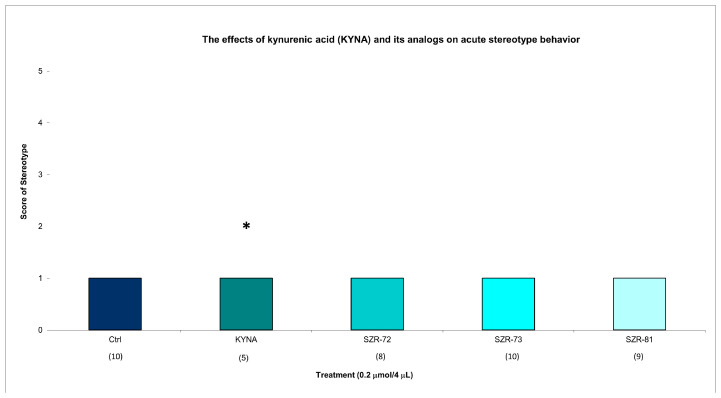
The effects of kynurenic acid and its analogs on acute stereotype behavior. Stereotype symptom scores of KYNA treatment, 0.2 µmol/4 µL KYNA vs. control (Ctrl) group (*p* = 0.014). We show the number of animals and the score categories. The level of significance and the number of animals in groups * *p* < 0.05; N(Ctrl) = 10, N(0.2 µmol/4 µL Kyna) = 10, N(0.2 µmol/4 µL SZR-72) = 10, N(0.2 µmol/4 µL SZR-73) = 10, N(0.2 µmol/4 µL SZR-81) = 10. The statistical analysis was a test of Normality with the Kolmogorov–Smirnov post hoc test and Homogeneity of variances with the Levene test and nonparametrical Kruskal–Wallis test. We show the number of animals in brackets.

KYNA elicited minimal stereotype behavior in a significantly lower number of animals (score 1 in five animals) at 15 min post injection, while all groups showed minimal stereotypic behavior invariably at the same dose and timepoint. KYNA nor KYNA analogs induced minimal stereotype behaviors (score 1 in 10 animals) at 45 min post injection (Figure 3, Table 2). By 45 min, KYNA-associated motor deficits subsided (ataxia: score 0 in 10 animals; stereotype: score 1 in 10 animals), aligning with its transient pharmacodynamic profile (Table 2). These findings underscore KYNA analogs’ improved motor safety profiles. For instance, SZR-73-treated mice exhibited near-baseline ataxia scores (score 0 in 10 animals) and negligible stereotype (score 1 in 10 animals), which are comparable to vehicle controls.

**Figure 4:**
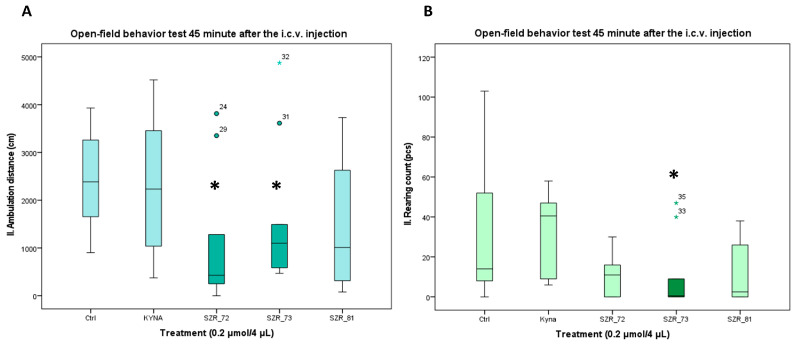
Open-field test 45 min after the i.c.v. injection. (**A**) Ambulation distance. We found a significant difference between the 0.2 µmol/4 µL SZR-72 vs. the 0.2 µmol/4 µL Kyna (*p* = 0.046) and the 0.2 µmol/4 µL SZR-72 and SZR_73 vs. control (Ctrl) group (*p* = 0.046). (**B**) Rearing count. We found a significant difference between the 0.2 µmol/4 µL SZR-73 vs. the 0.2 µmol/4 µL KYNA and control (Ctrl) group (*p* = 0.02). We show the data median ± SD. The level of significance and the number of animals in groups * *p* < 0.05; N(Ctrl) = 10, N(0.2 µmol/4 µL KYNA) = 10, N(0.2 µmol/4 µL SZR-72) = 10, N(0.2 µmol/4 µL SZR-73) = 10, N(0.2 µmol/4 µL SZR-81) = 10. The statistical analyses included a Normality test using the Kolmogorov–Smirnov post hoc test, an assessment of Homogeneity of variances via the Levene test, and the nonparametric Kruskal–Wallis test. KYNA: kynurenic acid; SZR-72: *N*-(2-(dimethylamino)ethyl)-4-hydroxyquinoline-2-carboxamide hydrochloride; SZR-73: *N*-(3-(dimethylamino)propyl)-4-hydroxyquinoline-2-carboxamide hydrochloride; SZR-81: 4-hydroxy-*N*-(2-(pyrrolidin-1-yl)ethyl)quinoline-2-carboxamide hydrochloride.

This study revealed transient motor effects following acute administration of KYNA and its analogs. At 15 min post injection, KYNA elicited significantly increased ataxia and significantly decreased stereotype compared to vehicle controls. This decrease was due to the fact that 5 out of 10 animals had ataxia and the other 5 had grade 1 stereotype, whereas none of the analogs (SZR-72, SZR-73, and SZR-81) induced statistically significant motor impairments at this time point. However, by 45 min, the differences in ataxia and stereotype scores between KYNA-treated and control groups diminished, with no significant intergroup variations observed (Figure 1, Table 2). This loss of significance suggests that KYNA’s motor-disrupting effects are short-lived, resolving within the testing window. In contrast, the analogs consistently avoided inducing significantly measurable stereotype or ataxia at both 15 and 45 min intervals, indicating a more favorable acute motor profile. Notably, open-field ambulation and rearing behaviors remained unaffected across all groups at 15 min, reinforcing the fact that locomotor activity was not broadly disrupted (Figure 4, Table 3). The transient nature of KYNA’s effects underscores its acute pharmacodynamic action, potentially linked to rapid metabolism or receptor desensitization. These findings highlight a critical temporal dimension in evaluating KYNA-related compounds, emphasizing that behavioral outcomes may vary substantially across time points. The analogs’ lack of significant motor effects, even at matched doses, positions them as candidates with reduced acute side effect liabilities compared to the parent compound.

### 3.2. Open-Field Activity: Delayed Modulation by Analogs

Open-field assays corroborated these trends. At 15 min post injection, KYNA and its analogs had no significant difference in locomotion distance or rearing count compared to vehicle control; however, at 45 min post injection, SZR-72 and SZR-73 significantly reduced locomotion distance, with only SZR-73 showing a significant reduction in rearing count (Figure 4A,B, Table 3). The open-field assessments revealed time-dependent behavioral modulation by KYNA analogs SZR-72 and SZR-73. At 15 min post injection, no significant differences in ambulation distance or rearing behavior were observed among treatment groups. However, by 45 min, both SZR-72 and SZR-73 elicited a marked decrease in ambulation and SZR-73 reduced rearing frequency, respectively. However, KYNA and SZR-81 showed no such effects (Figure 4, Table 3). Notably, KYNA-treated mice exhibited no significant locomotor or rearing changes at either time point, indicating its limited influence on open-field activity under these conditions. The delayed emergence of behavioral effects for SZR-72/SZR-73 may reflect slower pharmacokinetics, prolonged receptor engagement, or metabolite-mediated actions. The findings underscore the importance of temporal analysis in characterizing compound effects, as critical behavioral differences manifested only after prolonged observation. SZR-72 and SZR-73’s delayed modulation of ambulation and rearing positions them as candidates for conditions requiring time-dependent neurobehavioral activation, potentially avoiding sedation risks associated with immediate-onset stimulants.

**Figure 5:**
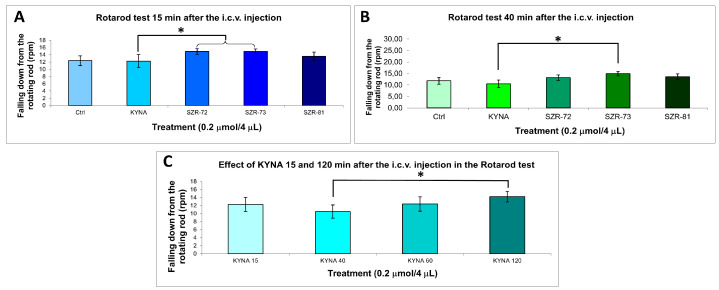
Rotarod behavior test. (**A**) Falling down from the rotating rod 15 min after the i.c.v. injection. We found significant difference the 0.2 µmol/4 µL SZR-72 (*p* = 0.032) and 0.2 µmol/4 µL SZR-73 (*p* = 0.030) vs. the 0.2 µmol/4 µL KYNA group. (**B**) Falling down from the rotating rod 40 min after the i.c.v. injection. We found significant difference the 0.2 µmol/4 µL SZR-73 vs. the 0.2 µmol/4 µL KYNA group (*p* = 0.018). (**C**) Falling down from the rotating rod 15–120 min after the i.c.v. injection. We found significant difference the 0.2 µmol/4 µL KYNA 40 min vs. the 0.2 µmol/4 µL KYNA 120 min group (*p* = 0.049). We show the data mean ± SEM. The level of significance and the number of animals in groups * *p* < 0.05; N(Ctrl) = 7, N(0.2 µmol/4 µL KYNA) = 10, N(0.2 µmol/4 µL SZR-72) = 10, N(0.2 µmol/4 µL SZR-73) = 10, N(0.2 µmol/4 µL SZR-81) = 10. The statistical tests used were the Kolmogorov–Smirnov post hoc test for normality, the Levene test for variance Homogeneity, and the one-way ANOVA with LSD post hoc test. RPM: revolution per minute; KYNA: kynurenic acid; SZR-72: *N*-(2-(dimethylamino)ethyl)-4-hydroxyquinoline-2-carboxamide hydrochloride; SZR-73: *N*-(3-(dimethylamino)propyl)-4-hydroxyquinoline-2-carboxamide hydrochloride; SZR-81: 4-hydroxy-*N*-(2-(pyrrolidin-1-yl)ethyl)quinoline-2-carboxamide hydrochloride.

Rotarod performance further validated the potential of a KYNA analog: SZR-73 and SZR-72 significantly increased latency to fall at 15 min and SZR-73 at 40 min compared to KYNA, whereas other analogs maintained baseline coordination (Figure 5, Table 4). The KYNA at 120 min significantly increased the latency to fall compared the KYNA at 40 min. These results collectively demonstrate that the structural optimization of KYNA successfully uncouples neuromodulatory potential from acute motor toxicity, positioning SZR-73 as a lead candidate for further therapeutic exploration.

### 3.3. Summary of Key Findings

This study demonstrated distinct time-dependent behavioral profiles for KYNA analogs, with marked reductions in motor side effects compared to the parent compound. While KYNA induced transient ataxia and decreased stereotype at 15 min significantly, these effects resolved by 45 min, underscoring its acute but short-lived pharmacodynamic action. In contrast, analogs SZR-72 and SZR-73 avoided early motor impairments and instead elicited delayed behavioral modulation, with significant decreases in open-field ambulation and rearing at 45 min, suggesting time-dependent neuroinhibitory or anxiolytic properties. SZR-73 emerged as a standout candidate, combining the delayed exploratory behavior decrease with superior rotarod performance, maintaining motor coordination comparable to controls. SZR-81 showed intermediate effects, mitigating KYNA’s motor deficits but lacking the delayed activation seen in SZR-72/SZR-73. Crucially, all analogs avoided the acute ataxia or sedation linked to KYNA, highlighting their refined safety profiles. These findings reveal dissociation between analog-specific effects: SZR-72/SZR-73 drive delayed behavioral activation, while SZR-73 uniquely preserved motor function. The time- and compound-dependent outcomes emphasize the importance of structural optimization in KYNA derivatives to balance therapeutic efficacy and tolerability. Collectively, the data position SZR-73 as a lead candidate for neurological disorders requiring sustained motor coordination and controlled behavioral modulation, offering a pathway to circumvent KYNA’s dose-limiting side effects (Table 5).

## 4. Discussion

This study reveals that SZR-73, a structural analog of KYNA, uniquely retains neuromodulatory properties while circumventing KYNA’s acute motor deficits, positioning it as a promising therapeutic candidate [59]. Unlike KYNA, which induced transient ataxia and stereotypic behaviors, SZR-73 exhibited minimal motor disruption at equivalent doses. Our choice of kynurenic acid as a positive control is grounded in its well-documented behavioral effects, including ataxia, as shown in early pivotal studies [reference]. These findings not only establish its functional relevance for behavioral assessments, but also justify its use in probing structure–activity relationships among kynurenine pathway metabolites. This divergence likely stems from strategic structural modifications—such as side chain alterations or functional group substitutions—that enhance receptor selectivity or reduce off-target interactions with motor-regulating pathways such as dopaminergic or cerebellar neural pathways. For instance, SZR-73’s preserved efficacy in reducing neurotoxic excitotoxicity or modulating glutamate receptors may derive from retained engagement with the KYN pathway, while its modified structure minimizes binding to receptors linked to motor dysfunction. The self-limiting nature of KYNA-induced ataxia most likely reflects rapid pharmacokinetic–pharmacodynamic dissociation. Microdialysis studies show that KYNA concentrations in the cerebellum drop steeply within 30 min, presumably via active efflux transporters, while NMDA and α7-nicotinic receptors undergo fast desensitization. Together, these processes curtail motor impairment despite KYNA’s metabolic stability. The newly synthesized analogs add a further layer of safety: their polar side chains accelerate clearance and weaken binding to motor-critical receptors, explaining why neither KYNA nor its derivatives produce ataxia at the 45 min mark. These considerations also argue against enzymatic degradation as the primary driver and instead highlight transporter-mediated redistribution and receptor kinetics as key determinants of the observed time course. Such dissociation between therapeutic and adverse effects suggests that SZR-73’s pharmacodynamic profile prioritizes potential neuroprotection over motor interference, a critical advantage for treating disorders like epilepsy or SCZ, where KYNA’s side effects limit utility. However, mechanistic clarity remains elusive; this study does not identify specific molecular targets or metabolic pathways responsible for these differences. Furthermore, findings are confined to acute and subacute single-dose paradigms in healthy mice, leaving chronic safety and disease-relevant efficacy unverified. Future work should employ knockout models, receptor-binding assays, or electrophysiology to pinpoint mechanisms, while validating SZR-73’s benefits in pathological contexts. These insights underscore the potential of structure-guided optimization to refine neuromodulators, balancing efficacy with tolerability in translational neuroscience. The biological hypothesis driving the present work is that KYNA’s motor-impairing liabilities arise from structural elements that can be fine-tuned without abolishing its neuroprotective core. By contrasting KYNA with analogs that differ only in side chain length or basicity, we show that a three-carbon dimethyl-propyl amide (SZR-73) decouples beneficial glutamatergic modulation from cerebellar-mediated ataxia. This finding addresses a long-standing translational bottleneck in the kynurenine field—how to retain pathway-specific neuroprotection while eliminating behavioral toxicity—and establishes a testable framework for future analog design and disease model validation.

This study highlights the promising therapeutic applications of KYNA analogs, particularly for SCZ and PD, by addressing critical limitations of native KYNA. In SCZ-relevant paradigms, SZR-72 and SZR-73 retained decreased stereotypic behaviors—a proxy for antipsychotic efficacy—without inducing sedation, suggesting the selective modulation of glutamatergic or dopaminergic hyperactivity implicated in psychosis. This aligns with their structural modifications, such as optimized side chains, which may enhance receptor specificity to disrupt pathological circuits while sparing motor function [96,97,98]. For PD, SZR-73 demonstrated improved rotarod performance, indicating preserved motor coordination and reduced ataxia (Figure 5A,B, Table 4), likely due to altered interactions with cerebellar or mitochondrial pathways critical for movement. These findings imply that KYNA analogs can dissociate neuroprotective benefits, including excitotoxicity reduction from motor side effects, a pivotal advance for disorders requiring long-term neuromodulation. However, the translational promise is constrained by the study’s acute and subacute single-dose design in healthy mice, which cannot model the chronic neurodegeneration or dopamine depletion seen in PD, nor complex SCZ pathophysiology [99,100,101]. Mechanistic ambiguities—such as exact receptor targets or metabolic stability—further cloud clinical extrapolation [99,100,101]. Future studies must prioritize disease models such as α-synucleinopathy or the NMDA hypofunction paradigm and chronic dosing to assess sustained efficacy and safety [99,100,101]. By resolving these gaps, KYNA analogs could emerge as dual-action agents, mitigating psychiatric symptoms via pathway-specific modulation while preserving motor integrity, ultimately bridging the divide between preclinical innovation and patient-centered therapeutic outcomes [99,100,101].

The structural modifications of KYNA analogs—SZR-72, SZR-73, and SZR-81—appear to drive distinct pharmacodynamic profiles, potentially by altering BBB penetration or receptor affinity. For instance, SZR-73’s reduced motor side effects such as minimal ataxia and preserved rotarod performance may stem from optimized BBB permeability, enabling selective CNS engagement without the broad disruption of motor circuits. Conversely, SZR-72’s efficacy in reducing stereotypic behaviors suggests enhanced affinity for the glutamatergic or dopaminergic receptors implicated in SCZ, while its structural tweaks such as bulkier side chains might limit off-target binding to cerebellar pathways. SZR-81’s improved motor coordination, meanwhile, could reflect the preferential modulation of mitochondrial or synaptic plasticity pathways over receptors linked to sedation. These divergences imply subtle structural changes (e.g., functional group substitutions, side chain additions), fine-tuned receptor interactions, or metabolic stability, decoupling therapeutic actions from adverse effects [30,62,102,103,104]. However, this study lacks direct evidence of BBB kinetics or receptor-binding specificity, leaving mechanistic explanations speculative. The acute behavioral differences observed—such as SZR-73’s delayed effects at 45 min—may also reflect variable pharmacokinetics, with slower CNS uptake or prolonged receptor occupancy. While these profiles underscore the potential of medicinal chemistry to refine neuromodulators, the absence of mechanistic data, including receptor autoradiography, pharmacokinetic assays, etc., limits translational certainty [62,105,106,107,108]. Future studies must resolve whether structural modifications primarily influence target engagement, BBB dynamics, or metabolic pathways, a critical step toward the rational design of analogs with tailored therapeutic windows for complex neurological disorders. For instance, recent reviews highlight alternative pathways for neuroinflammatory modulation, underscoring the importance of diversified therapeutic innovation alongside the structural optimization of KYNA derivatives [38].

While this study provides critical insights into the acute behavioral profiles of KYNA analogs, several limitations temper translational optimism. First, the findings are derived from single-dose acute experiments in healthy mice, precluding conclusions about chronic dosing effects, cumulative toxicity, or therapeutic durability, factors pivotal for disorders requiring sustained treatment, such as SCZ or PD. Second, the mechanistic ambiguity surrounding the analogs’ actions remains unresolved: structural modifications (e.g., SZR-73’s side chain) are hypothesized to alter receptor affinity, BBB penetration, or metabolic pathways, but direct evidence (e.g., receptor-binding assays, pharmacokinetic profiling) is absent. Without clarifying molecular targets, off-site interactions or unforeseen toxicities in humans cannot be ruled out. Third, the lack of disease model validation limits clinical relevance. For instance, SZR-73’s reduced motor deficits in healthy mice may not extrapolate to PD models with dopaminergic degeneration or SCZ models featuring glutamatergic hypofunction, where compensatory pathways could alter drug responses. Similarly, neuroinflammatory or aging-related changes in disease states might modulate analog efficacy or safety. These gaps underscore the preliminary nature of the findings and the need for caution in interpreting therapeutic potential. Future studies must prioritize mechanistic investigations (e.g., receptor autoradiography, metabolomics) and validation in pathophysiological contexts to confirm whether structural refinements truly decouple therapeutic benefits from adverse effects. Until then, the analogs’ promise remains provisional, anchored to idealized preclinical conditions rather than the complexity of human neuropsychiatric disorders.

The translational promise of KYNA analogs hinges on addressing critical gaps between preclinical observations and clinical realities [27,108,109,110,111]. While acute studies in healthy mice highlight reduced motor side effects and retained neuromodulatory activity, chronic toxicity studies are urgently needed to evaluate long-term safety, cumulative dosing effects, and potential withdrawal or tolerance, issues paramount for disorders like SCZ or PD requiring lifelong therapy [112,113,114]. Furthermore, receptor-binding assays are essential to resolve mechanistic ambiguities; structural modifications in SZR-73 or SZR-81 may alter affinity for NMDA, α7-nACh, or AHR receptors, but without empirical validation, off-target interactions or interspecies variability in drug metabolism remain unaddressed [115,116,117,118,119]. Equally pressing is the need to test analogs in comorbid disease models, such as PD with depression or SCZ with cognitive deficits, where overlapping pathologies could modulate drug efficacy or safety [120,121,122,123]. For instance, neuroinflammation in AD or dopamine depletion in PD might amplify or negate the analog effects observed in healthy mice. Additionally, pharmacokinetic profiling, including BBB penetration and metabolite stability, must complement behavioral data to predict human dosing regimens [124,125,126,127]. A further limitation is the lack of pharmacokinetic data describing the elimination of KYNA and its structural analogs from the brain to the periphery. Because our paradigm relied on acute intracerebroventricular administration and a short observation window, no plasma or cerebrospinal fluid samples were obtained. Consequently, the current dataset cannot determine whether the analogs are cleared more rapidly than native KYNA or whether active efflux transporters contribute to their transient behavioral profile. Future studies should integrate serial plasma sampling, the determination of brain-to-plasma ratios, and transporter blockade experiments to quantify peripheral clearance and establish exposure–response relationships, thereby informing dose selection for chronic and systemic delivery. These steps are not merely incremental, but foundational: they transform structure–activity correlations into actionable insights for clinical trial design. Only by integrating chronic safety data, mechanistic clarity, and disease complexity can KYNA analogs transition from promising preclinical candidates to viable therapies, ensuring their benefits withstand the multifaceted challenges of human neuropsychiatric care [128,129].

To advance SZR-73 as a lead KYNA analog, future research must prioritize three strategic pillars: pharmacogenomics, nanodelivery systems, and human neuron assays. Pharmacogenomic studies could elucidate genetic variants influencing SZR-73’s efficacy or toxicity, enabling personalized dosing strategies for neuropsychiatric disorders with diverse genetic underpinnings (e.g., SCZ-linked *COMT* polymorphisms) [130,131,132,133]. Concurrently, nanodelivery systems—such as lipid nanoparticles or polymer-based carriers—should be explored to enhance SZR-73’s BBB penetration, prolong half-life, or target specific brain regions (e.g., striatum in PD), potentially reducing systemic exposure and off-target effects [134,135,136,137,138]. Integrating these systems with real-time biodistribution imaging could refine delivery precision. Finally, human neuron assays—using iPSC-derived neurons or 3D organoids—are critical to validate SZR-73’s effects on synaptic plasticity, neuroinflammation, or excitotoxicity in human-relevant contexts, circumventing the limitations of rodent models [139,140,141,142,143]. For example, testing SZR-73 in dopaminergic neurons from PD patients or cortical circuits from SCZ cohorts could reveal disease-specific synergies or vulnerabilities. Together, these approaches would bridge mechanistic insights from acute mouse studies to human pathophysiology, addressing gaps in target specificity, metabolic stability, and interspecies translatability. By coupling structural innovation with translational tools, SZR-73 could evolve from a preclinical candidate to a precision therapeutic, tailored to the genetic, anatomical, and molecular complexities of neurological and psychiatric diseases.

Building on our comparative analysis of SZR-72/73/81 and legacy KYNA data, we distilled four predictive SAR principles that can guide the design of next-generation KYNA analogs for movement and psychotic disorders. First, three-carbon dimethyl-amide side chains (as exemplified by SZR-73) preserve neuroprotective glutamatergic modulation while abrogating cerebellar ataxia, whereas bulkier more basic side chains (such as those in SZR-72) preferentially damp stereotypy via dopaminergic and glutamatergic hyperactivity suppression. Second, by integrating published 3D-QSAR and molecular docking data for NMDA and α7-nicotinic antagonists with our behavioral profiles, we constructed a heuristic table that predicts whether future analogs will favor an anti-Parkinson’s or anti-schizophrenia profile based on key structural motifs. Third, we propose cheminformatic filters—namely clogP ≤ 2.5 and H-bond donors ≤ 2—to ensure robust blood–brain barrier penetration without off-target motor-circuit spill-over, as supported by our BBB permeability discussion. Finally, we outline an in silico screening workflow (LigandScout pharmacophore modeling → AutoDock Vina docking → ADMET Predictor assessment) that can be readily adopted by other researchers to rank novel KYNA scaffolds prior to synthesis and in vivo validation. To facilitate translation, we recommend that all lead candidates be benchmarked using our rotarod and open-field pipelines alongside microdialysis-based pharmacokinetics, allowing for the iterative refinement of efficacy–toxicity indices before embarking on rigorous disease model trials and, ultimately, clinical assessment in downstream phases.

## 5. Conclusions

The structural optimization of KYNA into its analogs—notably SZR-73—demonstrates a pivotal success in mitigating KYNA’s dose-limiting motor side effects, such as acute ataxia and stereotypic behaviors, while preserving its potential neuroprotective and neuromodulatory potential. SZR-73 emerges as a lead candidate, uniquely balancing therapeutic efficacy with motor safety, as evidenced by its reduced rotarod deficits and retained anxiolytic properties, likely due to strategic modifications like side chain alterations that enhance receptor selectivity or BBB dynamics. However, this promise remains provisional without resolving key gaps: mechanistic studies to pinpoint molecular targets, chronic dosing trials to assess long-term safety, and validation in disease models replicating neurodegeneration or psychiatric pathophysiology. Bridging these gaps demands interdisciplinary collaboration, integrating medicinal chemistry, systems pharmacology, and clinical neuropsychiatry to refine pharmacokinetics, optimize delivery systems, and validate outcomes in human-derived models. By uniting these efforts, the field can advance KYNA-based therapies beyond preclinical promise into clinical trials, ensuring that structural innovation translates into safer effective treatments for disorders like PD or SCZ. The journey from bench to bedside hinges on sustained collaborative rigor, a call to action for researchers and clinicians alike to transform molecular potential into patient-centered breakthroughs [144].

## Figures and Tables

**Table 1:** Scoring criteria for ataxia and stereotype behaviors. This table outlines the behavioral scoring system used to evaluate the severity of motor impairments and stereotyped movements. Ataxia scores range from mild coordination issues to complete immobility, while stereotype scores capture increasing intensity and complexity of repetitive or abnormal behaviors. This system allows for standardized graded assessment in experimental models.

Behavior		Score
Ataxia	Awkward and jerky movements	1
	Stumbling or awkward posture	2
	Falling	3
	Inability to move beyond a small area or support weight on the stomach or haunches	4
	Inability to move, except for twitching movements	5
Stereotype	Sniffing, grooming, and rearing, reciprocal forepaw treading or undirected head movement	1
	Backward walking, head weaving, circling behavior	2
	Continuous head weaving, circling, or backward walking	3
	Dyskinetic extensions or flexion of the limbs	4
	Head and neck or weaving greater than four	5

**Table 2:** Acute and subacute effects of test compounds on ataxia and stereotype scores. Behavioral scores recorded at 15 and 45 min after the administration of control or kynurenic acid analogs. Ataxia and stereotype were assessed using a standardized scale, with the number of animals exhibiting each score indicated in parentheses. Data reflect both immediate (acute) and delayed (subacute) motor and behavioral responses.

Compounds	Ataxia Score (Number of Animals)		Stereotype Score (Number of Animals)	
	15 min	45 min	15 min	45 min
Control	0 (10)	0 (10)	1 (10)	1 (10)
KYNA	3 (2), 4 (3) ^1^	0 (10)	1 (5) ^1^	1 (10)
SZR-72	4 (1), 5 (1)	0 (10)	1 (8)	1 (10)
SZR-73	0 (10)	0 (10)	1 (10)	1 (10)
SZR-81	1 (1)	0 (10)	1 (9)	1 (10)

^1^ *p* < 0.05 vs. KYNA. We show the number of animals/groups in brackets. KYNA: kynurenic acid; SZR-72: *N*-(2-(dimethylamino)ethyl)-4-hydroxyquinoline-2-carboxamide hydrochloride; SZR-73: *N*-(3-(dimethylamino)propyl)-4-hydroxyquinoline-2-carboxamide hydrochloride; SZR-81: 4-hydroxy-*N*-(2-(pyrrolidin-1-yl)ethyl)quinoline-2-carboxamide hydrochloride.

**Table 3:** Effects of kynurenic acid and analogs on locomotor and exploratory activity in the open-field test. Ambulation distance and rearing counts were measured at 15 and 45 min post treatment to assess locomotor and exploratory behavior.

Compounds	Ambulation Distance		Rearing Count	
	15 min	45 min	15 min	45 min
Control	3372.3 ± 441.925 (10)	2423.3 ± 328.405 (10)	38.6 ± 11.197 (10)	28.4 ± 10.341 (10)
KYNA	3085.6 ± 505.334 (10)	2278.6 ± 439.742 (10)	35.0 ± 9.084 (10)	32.8 ± 6.622 (10)
SZR-72	1699.7 ± 612.203 (10)	1081.7 ± 435.542 (10) ^1,2^	18.7 ± 6.762 (10)	10.2 ± 3.133 (10)
SZR-73	2224.9 ± 544.970 (10)	1600.6 ± 462.407 (10) ^1^	11.5 ± 5.807 (10)	9.8 ± 5.707 (10) ^1,2^
SZR-81	1794.0 ± 499.207 (10)	1397.7 ± 389.917 (10)	23.5 ± 9.653 (10)	12.1 ± 4.836 (10)

^1^ *p* < 0.05 vs. control, ^2^ *p* < 0.05 vs. KYNA. We show the number of animals/groups in brackets. KYNA: kynurenic acid; SZR-72: *N*-(2-(dimethylamino)ethyl)-4-hydroxyquinoline-2-carboxamide hydrochloride; SZR-73: *N*-(3-(dimethylamino)propyl)-4-hydroxyquinoline-2-carboxamide hydrochloride; SZR-81: *N*-(2-(pyrrolidin-1-yl)ethyl)-4-hydroxyquinoline-2-carboxamide hydrochloride.

**Table 4:** Effects of kynurenic acid and analogues on motor coordination in the rotarod test. Latency to fall was recorded at 15 to 120 min post treatment using an accelerating rod to assess motor coordination and balance.

Compounds	Latency to Fall (RPM)	
	15 min	40 min
Control	70.143 ± 15.322 (7)	40,777 ± 15.412 (7)
KYNA	58.590 ± 20.559 (10)	50.444 ± 15.952 (10)
SZR-72	76.840 ± 11.729 (10) ^1^	51.448 ± 16.269 (10)
SZR-73	77.600 ± 9.279 (10) ^1^	45.284 ± 14.320 (10) ^1^
SZR-81	64.420 ± 13.238 (10)	51.581 ± 16.311 (10)

^1^ *p* < 0.05 vs. KYNA. We show the number of animals/groups in brackets. RPM: revolution per minute; KYNA: kynurenic acid; SZR-72: *N*-(2-(dimethylamino)ethyl)-4-hydroxyquinoline-2-carboxamide hydrochloride; SZR-73: *N*-(3-(dimethylamino)propyl)-4-hydroxyquinoline-2-carboxamide hydrochloride; SZR-81: 4-hydroxy-*N*-(2-(pyrrolidin-1-yl)ethyl)quinoline-2-carboxamide hydrochloride.

**Table 5:** Therapeutic profile matrix of kynurenic acid (KYNA) analogs.

Analog	Key Structural Motif	Rotarod (Ataxia)	Open-Field Activity	Stereotype	Predicted Indication
KYNA	Native quinoline carboxylate	Impaired at 15 min; recovers by 45 min	Early hypoactivity, resolves	Mild or absent	Limited (motor toxicity)
SZR-72	Bulkier tertiary amine side chain	No impairment	Normal locomotion	Reduced hyperlocomotion	Anti-schizophrenia
SZR-73	Three-carbon dimethyl-amide side chain	No impairment	Mild, therapeutically desirable hypoactivity	None detected	Anti-Parkinson
SZR-81	Methyl-ester substituted side chain	No impairment	Neutral	Moderate	Neuroprotective/mixed

## Data Availability

The original contributions presented in this study are included in the article. Further inquiries can be directed to the corresponding authors.

## Abbreviations

The following abbreviations are used in this manuscript:

AD	Alzheimer’s disease
BBB	blood–brain barrier
KYN	kynurenine
KYNA	kynurenic acid
NMDA	N-methyl-D-aspartate
PD	Parkinson’s disease
SCZ	schizophrenia

## References

[1] Grezenko H., Rodoshi Z.N., Mimms C.S., Ahmed M., Sabani A., Hlaing M.S., Batu B.J., Hundesa M.I., Ayalew B.D., Shehryar A. (2024). From Alzheimer’s Disease to Anxiety, Epilepsy to Schizophrenia: A Comprehensive Dive Into Neuro-Psychiatric Disorders. Cureus.

[2] Holper L., Ben-Shachar D., Mann J.J. (2019). Multivariate meta-analyses of mitochondrial complex I and IV in major depressive disorder, bipolar disorder, schizophrenia, Alzheimer disease, and Parkinson disease. Neuropsychopharmacology.

[3] Berger T., Lee H., Young A.H., Aarsland D., Thuret S. (2020). Adult hippocampal neurogenesis in major depressive disorder and Alzheimer’s disease. Trends Mol. Med..

[4] Borumandnia N., Majd H.A., Doosti H., Olazadeh K. (2022). The trend analysis of neurological disorders as major causes of death and disability according to human development, 1990–2019. Environ. Sci. Pollut. Res..

[5] Cadeddu C., Ianuale C., Lindert J. (2015). Public mental health. A Systematic Review of Key Issues in Public Health.

[6] Tanaka M., Vécsei L. (2024). Revolutionizing our understanding of Parkinson’s disease: Dr. Heinz Reichmann’s pioneering research and future research direction. J. Neural Transm..

[7] Al-Mubarak B., Ahmed Nour M., Schumacher-Schuh A., Bandres-Ciga S. (2022). Globalizing Research toward Diverse Representation in Alzheimer’s and Parkinson’s Disease. Ann. Neurol..

[8] Wang Z., Wang Q., Li S., Li X.-J., Yang W., He D. (2023). Microglial autophagy in Alzheimer’s disease and Parkinson’s disease. Front. Aging Neurosci..

[9] Rehm J., Shield K.D. (2019). Global burden of disease and the impact of mental and addictive disorders. Curr. Psychiatry Rep..

[10] Javaid S.F., Giebel C., Khan M.A., Hashim M.J. (2021). Epidemiology of Alzheimer’s disease and other dementias: Rising global burden and forecasted trends. F1000Research.

[11] Poalelungi A., Popescu B.O. (2013). Alzheimer’s disease–neurological or psychiatric disorder?. Rom. J. Neurol..

[12] Sadeghi I., Gispert J.D., Palumbo E., Muñoz-Aguirre M., Wucher V., D’Argenio V., Santpere G., Navarro A., Guigo R., Vilor-Tejedor N. (2022). Brain transcriptomic profiling reveals common alterations across neurodegenerative and psychiatric disorders. Comput. Struct. Biotechnol. J..

[13] Better M.A. (2023). Alzheimer’s disease facts and figures. Alzheimers Dement..

[14] Frankish H., Horton R. (2017). Prevention and management of dementia: A priority for public health. Lancet.

[15] Cheslow L., Snook A.E., Waldman S.A. (2024). Biomarkers for Managing Neurodegenerative Diseases. Biomolecules.

[16] de Lima E.P., Tanaka M., Lamas C.B., Quesada K., Detregiachi C.R.P., Araújo A.C., Guiguer E.L., Catharin V.M.C.S., de Castro M.V.M., Junior E.B. (2024). Vascular impairment, muscle atrophy, and cognitive decline: Critical age-related conditions. Biomedicines.

[17] Márquez F., Yassa M.A. (2019). Neuroimaging biomarkers for Alzheimer’s disease. Mol. Neurodegener..

[18] Chen-Plotkin A.S. (2014). Unbiased approaches to biomarker discovery in neurodegenerative diseases. Neuron.

[19] Lang A.E., Espay A.J. (2018). Disease modification in Parkinson’s disease: Current approaches, challenges, and future considerations. Mov. Disord..

[20] Annus Á., Tömösi F., Rárosi F., Fehér E., Janáky T., Kecskeméti G., Toldi J., Klivényi P., Sztriha L., Vécsei L. (2021). Kynurenic acid and kynurenine aminotransferase are potential biomarkers of early neurological improvement after thrombolytic therapy: A pilot study. Adv. Clin. Exp. Med..

[21] Török N., Tanaka M., Vécsei L. (2020). Searching for peripheral biomarkers in neurodegenerative diseases: The tryptophan-kynurenine metabolic pathway. Int. J. Mol. Sci..

[22] Pizarro-Galleguillos B.M., Kunert L., Brüggemann N., Prasuhn J. (2023). Neuroinflammation and mitochondrial dysfunction in parkinson’s disease: Connecting neuroimaging with pathophysiology. Antioxidants.

[23] Savitz J. (2020). The kynurenine pathway: A finger in every pie. Mol. Psychiatry.

[24] Lovelace M.D., Varney B., Sundaram G., Lennon M.J., Lim C.K., Jacobs K., Guillemin G.J., Brew B.J. (2017). Recent evidence for an expanded role of the kynurenine pathway of tryptophan metabolism in neurological diseases. Neuropharmacology.

[25] Behl T., Kaur I., Sehgal A., Singh S., Bhatia S., Al-Harrasi A., Zengin G., Bumbu A.G., Andronie-Cioara F.L., Nechifor A.C. (2021). The footprint of kynurenine pathway in neurodegeneration: Janus-faced role in Parkinson’s disorder and therapeutic implications. Int. J. Mol. Sci..

[26] Martín-Hernández D., Tendilla-Beltrán H., Madrigal J.L., García-Bueno B., Leza J.C., Caso J.R. (2019). Chronic mild stress alters kynurenine pathways changing the glutamate neurotransmission in frontal cortex of rats. Mol. Neurobiol..

[27] Szabo M., Lajkó N., Dulka K., Szatmári I., Fülöp F., Mihály A., Vécsei L., Gulya K. (2022). Kynurenic acid and its analog SZR104 exhibit strong antiinflammatory effects and alter the intracellular distribution and methylation patterns of H3 histones in immunochallenged microglia-enriched cultures of newborn rat brains. Int. J. Mol. Sci..

[28] Braidy N., Grant R. (2017). Kynurenine pathway metabolism and neuroinflammatory disease. Neural Regen. Res..

[29] Tanaka M., Szabó Á., Vécsei L. (2024). Redefining roles: A paradigm shift in tryptophan–kynurenine metabolism for innovative clinical applications. Int. J. Mol. Sci..

[30] Hilmas C., Pereira E.F., Alkondon M., Rassoulpour A., Schwarcz R., Albuquerque E.X. (2001). The brain metabolite kynurenic acid inhibits α7 nicotinic receptor activity and increases non-α7 nicotinic receptor expression: Physiopathological implications. J. Neurosci..

[31] Secci M.E., Mascia P., Sagheddu C., Beggiato S., Melis M., Borelli A.C., Tomasini M.C., Panlilio L.V., Schindler C.W., Tanda G. (2019). Astrocytic Mechanisms Involving Kynurenic Acid Control Δ 9-Tetrahydrocannabinol-Induced Increases in Glutamate Release in Brain Reward-Processing Areas. Mol. Neurobiol..

[32] Stone T., Behan W., Jones P., Darlington L., Smith R. (2001). The role of kynurenines in the production of neuronal death, and the neuroprotective effect of purines. J. Alzheimer’s Dis..

[33] Moroni F., Cozzi A., Sili M., Mannaioni G. (2012). Kynurenic acid: A metabolite with multiple actions and multiple targets in brain and periphery. J. Neural Transm..

[34] Mok M.S., Fricker A.-C., Weil A., Kew J.N. (2009). Electrophysiological characterisation of the actions of kynurenic acid at ligand-gated ion channels. Neuropharmacology.

[35] Alkondon M., Pereira E.F., Albuquerque E.X. (2011). Endogenous activation of nAChRs and NMDA receptors contributes to the excitability of CA1 stratum radiatum interneurons in rat hippocampal slices: Effects of kynurenic acid. Biochem. Pharmacol..

[36] Bratek-Gerej E., Ziembowicz A., Godlewski J., Salinska E. (2021). The mechanism of the neuroprotective effect of kynurenic acid in the experimental model of neonatal hypoxia–ischemia: The link to oxidative stress. Antioxidants.

[37] Tajti J., Majlath Z., Szok D., Csati A., Toldi J., Fulop F., Vecsei L. (2015). Novel kynurenic acid analogues in the treatment of migraine and neurodegenerative disorders: Preclinical studies and pharmaceutical design. Curr. Pharm. Des..

[38] Barbalho S.M., Leme Boaro B., da Silva Camarinha Oliveira J., Patočka J., Barbalho Lamas C., Tanaka M., Laurindo L.F. (2025). Molecular Mechanisms Underlying Neuroinflammation Intervention with Medicinal Plants: A Critical and Narrative Review of the Current Literature. Pharmaceuticals.

[39] Wirthgen E., Hoeflich A., Rebl A., Günther J. (2018). Kynurenic acid: The Janus-faced role of an immunomodulatory tryptophan metabolite and its link to pathological conditions. Front. Immunol..

[40] Kindler J., Lim C.K., Weickert C.S., Boerrigter D., Galletly C., Liu D., Jacobs K.R., Balzan R., Bruggemann J., O’Donnell M. (2020). Dysregulation of kynurenine metabolism is related to proinflammatory cytokines, attention, and prefrontal cortex volume in schizophrenia. Mol. Psychiatry.

[41] Stone T.W., Forrest C.M., Darlington L.G. (2012). Kynurenine pathway inhibition as a therapeutic strategy for neuroprotection. FEBS J..

[42] Mackay G., Forrest C., Stoy N., Christofides J., Egerton M., Stone T., Darlington L. (2006). Tryptophan metabolism and oxidative stress in patients with chronic brain injury. Eur. J. Neurol..

[43] Barbalho S.M., Laurindo L.F., de Oliveira Zanuso B., da Silva R.M.S., Gallerani Caglioni L., Nunes Junqueira de Moraes V.B.F., Fornari Laurindo L., Dogani Rodrigues V., da Silva Camarinha Oliveira J., Beluce M.E. (2025). AdipoRon’s Impact on Alzheimer’s Disease—A Systematic Review and Meta-Analysis. Int. J. Mol. Sci..

[44] Lim C.K., Fernandez-Gomez F.J., Braidy N., Estrada C., Costa C., Costa S., Bessede A., Fernandez-Villalba E., Zinger A., Herrero M.T. (2017). Involvement of the kynurenine pathway in the pathogenesis of Parkinson’s disease. Prog. Neurobiol..

[45] Sorgdrager F.J., Vermeiren Y., Van Faassen M., Van Der Ley C., Nollen E.A., Kema I.P., De Deyn P.P. (2019). Age-and disease-specific changes of the kynurenine pathway in Parkinson’s and Alzheimer’s disease. J. Neurochem..

[46] Sharma R., Razdan K., Bansal Y., Kuhad A. (2018). Rollercoaster ride of kynurenines: Steering the wheel towards neuroprotection in Alzheimer’s disease. Expert. Opin. Ther. Targets.

[47] Ostapiuk A., Urbanska E.M. (2022). Kynurenic acid in neurodegenerative disorders—Unique neuroprotection or double-edged sword?. CNS Neurosci. Ther..

[48] Szabó Á., Galla Z., Spekker E., Szűcs M., Martos D., Takeda K., Ozaki K., Inoue H., Yamamoto S., Toldi J. (2025). Oxidative and Excitatory Neurotoxic Stresses in CRISPR/Cas9-Induced Kynurenine Aminotransferase Knockout Mice: A Novel Model for Despair-Based Depression and Post-Traumatic Stress Disorder. Front. Biosci..

[49] Martos D., Tuka B., Tanaka M., Vécsei L., Telegdy G. (2022). Memory enhancement with kynurenic acid and its mechanisms in neurotransmission. Biomedicines.

[50] Hunt C., e Cordeiro T.M., Suchting R., de Dios C., Leal V.A.C., Soares J.C., Dantzer R., Teixeira A.L., Selvaraj S. (2020). Effect of immune activation on the kynurenine pathway and depression symptoms–a systematic review and meta-analysis. Neurosci. Biobehav. Rev..

[51] Leclercq S., Schwarz M., Delzenne N.M., Stärkel P., de Timary P. (2021). Alterations of kynurenine pathway in alcohol use disorder and abstinence: A link with gut microbiota, peripheral inflammation and psychological symptoms. Transl. Psychiatry.

[52] Kloc R., Urbanska E.M. (2024). Memantine and the Kynurenine Pathway in the Brain: Selective Targeting of Kynurenic Acid in the Rat Cerebral Cortex. Cells.

[53] Klein C., Patte-Mensah C., Taleb O., Bourguignon J.-J., Schmitt M., Bihel F., Maitre M., Mensah-Nyagan A.G. (2013). The neuroprotector kynurenic acid increases neuronal cell survival through neprilysin induction. Neuropharmacology.

[54] Lőrinczi B., Szatmári I. (2021). KYNA derivatives with modified skeleton; hydroxyquinolines with potential neuroprotective effect. Int. J. Mol. Sci..

[55] Foster A.C., Vezzani A., French E.D., Schwarcz R. (1984). Kynurenic acid blocks neurotoxicity and seizures induced in rats by the related brain metabolite quinolinic acid. Neurosci. Lett..

[56] Zádori D., Nyiri G., Szőnyi A., Szatmári I., Fülöp F., Toldi J., Freund T.F., Vécsei L., Klivényi P. (2011). Neuroprotective effects of a novel kynurenic acid analogue in a transgenic mouse model of Huntington’s disease. J. Neural Transm..

[57] Phenis D., Vunck S.A., Valentini V., Arias H., Schwarcz R., Bruno J.P. (2020). Activation of alpha7 nicotinic and NMDA receptors is necessary for performance in a working memory task. Psychopharmacology.

[58] Pocivavsek A., Elmer G.I., Schwarcz R. (2019). Inhibition of kynurenine aminotransferase II attenuates hippocampus-dependent memory deficit in adult rats treated prenatally with kynurenine. Hippocampus.

[59] Martos D., Lőrinczi B., Szatmári I., Vécsei L., Tanaka M. (2024). The Impact of C-3 Side Chain Modifications on Kynurenic Acid: A Behavioral Analysis of Its Analogs in the Motor Domain. Int. J. Mol. Sci..

[60] Knyihar-Csillik E., Mihaly A., Krisztin-Peva B., Robotka H., Szatmari I., Fulop F., Toldi J., Csillik B., Vecsei L. (2008). The kynurenate analog SZR-72 prevents the nitroglycerol-induced increase of c-fos immunoreactivity in the rat caudal trigeminal nucleus: Comparative studies of the effects of SZR-72 and kynurenic acid. Neurosci. Res..

[61] Lőrinczi B., Csámpai A., Fülöp F., Szatmári I. (2020). Synthesis of New C-3 Substituted Kynurenic Acid Derivatives. Molecules.

[62] Fehér E., Szatmári I., Dudás T., Zalatnai A., Farkas T., Lőrinczi B., Fülöp F., Vécsei L., Toldi J. (2019). Structural Evaluation and Electrophysiological Effects of Some Kynurenic Acid Analogs. Molecules.

[63] Lőrinczi B., Csámpai A., Fülöp F., Szatmári I. (2020). Synthetic- and DFT modelling studies on regioselective modified Mannich reactions of hydroxy-KYNA derivatives. RSC Adv..

[64] Lapin I.P. (1982). Convulsant action of intracerebroventricularly administered l-kynurenine sulphate, quinolinic acid and other derivatives of succinic acid, and effects of amino acids: Structure-activity relationships. Neuropharmacology.

[65] Lapin I.P. (1978). Stimulant and convulsive effects of kynurenines injected into brain ventricles in mice. J. Neural Transm..

[66] Vécsei L., Beal M.F. (1990). Intracerebroventricular injection of kynurenic acid, but not kynurenine, induces ataxia and stereotyped behavior in rats. Brain Res. Bull..

[67] Lapin I.P., Prakhie I.B., Kiseleva I.P. (1982). Excitatory effects of kynurenine and its metabolites, amino acids and convulsants administered into brain ventricles: Differences between rats and mice. J. Neural. Transm..

[68] Kim D.H., Jung J.S., Moon Y.S., Song D.K. (2009). Central or peripheral norepinephrine depletion enhances MK-801-induced plasma corticosterone level in mice. Prog. Neuropsychopharmacol. Biol. Psychiatry.

[69] Olsson I.A., Hansen A.K., Sandøe P. (2007). Ethics and refinement in animal research. Science.

[70] McGinn R., Fergusson D.A., Stewart D.J., Kristof A.S., Barron C.C., Thebaud B., McIntyre L., Stacey D., Liepmann M., Dodelet-Devillers A. (2021). Surrogate Humane Endpoints in Small Animal Models of Acute Lung Injury: A Modified Delphi Consensus Study of Researchers and Laboratory Animal Veterinarians. Crit. Care Med..

[71] Zingarelli B., Coopersmith C.M., Drechsler S., Efron P., Marshall J.C., Moldawer L., Wiersinga W.J., Xiao X., Osuchowski M.F., Thiemermann C. (2019). Part I: Minimum Quality Threshold in Preclinical Sepsis Studies (MQTiPSS) for Study Design and Humane Modeling Endpoints. Shock.

[72] Nemzek J.A., Xiao H.Y., Minard A.E., Bolgos G.L., Remick D.G. (2004). Humane endpoints in shock research. Shock.

[73] Olfert E.D., Godson D.L. (2000). Humane endpoints for infectious disease animal models. Ilar J..

[74] Mei J., Banneke S., Lips J., Kuffner M.T.C., Hoffmann C.J., Dirnagl U., Endres M., Harms C., Emmrich J.V. (2019). Refining humane endpoints in mouse models of disease by systematic review and machine learning-based endpoint definition. Altex.

[75] Sturgeon R.D., Fessler R.G., Meltzer H.Y. (1979). Behavioral rating scales for assessing phencyclidine-induced locomotor activity, stereotyped behavior and ataxia in rats. Eur. J. Pharmacol..

[76] Contreras P.C. (1990). D-serine antagonized phencyclidine- and MK-801-induced stereotyped behavior and ataxia. Neuropharmacology.

[77] Castellani S., Adams P.M. (1981). Acute and chronic phencyclidine effects on locomotor activity, stereotypy and ataxia in rats. Eur. J. Pharmacol..

[78] Tanii Y., Nishikawa T., Hashimoto A., Takahashi K. (1994). Stereoselective antagonism by enantiomers of alanine and serine of phencyclidine-induced hyperactivity, stereotypy and ataxia in the rat. J. Pharmacol. Exp. Ther..

[79] Kraeuter A.K., Guest P.C., Sarnyai Z. (2019). The Open Field Test for Measuring Locomotor Activity and Anxiety-Like Behavior. Methods Mol. Biol..

[80] Jimenez J.A., McCoy E.S., Lee D.F., Zylka M.J. (2023). The open field assay is influenced by room temperature and by drugs that affect core body temperature. F1000Research.

[81] Seibenhener M.L., Wooten M.C. (2015). Use of the Open Field Maze to measure locomotor and anxiety-like behavior in mice. J. Vis. Exp..

[82] Widjaja J.H., Sloan D.C., Hauger J.A., Muntean B.S. (2023). Customizable Open-Source Rotating Rod (Rotarod) Enables Robust Low-Cost Assessment of Motor Performance in Mice. eNeuro.

[83] Shiotsuki H., Yoshimi K., Shimo Y., Funayama M., Takamatsu Y., Ikeda K., Takahashi R., Kitazawa S., Hattori N. (2010). A rotarod test for evaluation of motor skill learning. J. Neurosci. Methods.

[84] Deacon R.M. (2013). Measuring motor coordination in mice. J. Vis. Exp..

[85] Jones B.J., Roberts D.J. (1968). The quantiative measurement of motor inco-ordination in naive mice using an acelerating rotarod. J. Pharm. Pharmacol..

[86] Osmon K.J., Vyas M., Woodley E., Thompson P., Walia J.S. (2018). Battery of Behavioral Tests Assessing General Locomotion, Muscular Strength, and Coordination in Mice. J. Vis. Exp..

[87] Rustay N.R., Wahlsten D., Crabbe J.C. (2003). Influence of task parameters on rotarod performance and sensitivity to ethanol in mice. Behav. Brain Res..

[88] Scholz J., Niibori Y., Frankland P.W., Lerch J.P. (2015). Rotarod training in mice is associated with changes in brain structure observable with multimodal MRI. Neuroimage.

[89] Spink A.J., Tegelenbosch R.A., Buma M.O., Noldus L.P. (2001). The EthoVision video tracking system--a tool for behavioral phenotyping of transgenic mice. Physiol. Behav..

[90] Sturman O., von Ziegler L., Schläppi C., Akyol F., Privitera M., Slominski D., Grimm C., Thieren L., Zerbi V., Grewe B. (2020). Deep learning-based behavioral analysis reaches human accuracy and is capable of outperforming commercial solutions. Neuropsychopharmacology.

[91] Noldus L.P., Spink A.J., Tegelenbosch R.A. (2001). EthoVision: A versatile video tracking system for automation of behavioral experiments. Behav. Res. Methods Instrum. Comput..

[92] Grieco F., Bernstein B.J., Biemans B., Bikovski L., Burnett C.J., Cushman J.D., van Dam E.A., Fry S.A., Richmond-Hacham B., Homberg J.R. (2021). Measuring Behavior in the Home Cage: Study Design, Applications, Challenges, and Perspectives. Front. Behav. Neurosci..

[93] Stewart A.M., Grieco F., Tegelenbosch R.A., Kyzar E.J., Nguyen M., Kaluyeva A., Song C., Noldus L.P., Kalueff A.V. (2015). A novel 3D method of locomotor analysis in adult zebrafish: Implications for automated detection of CNS drug-evoked phenotypes. J. Neurosci. Methods.

[94] (1999). Recommendations for standards regarding preclinical neuroprotective and restorative drug development. Stroke.

[95] Henderson V.C., Kimmelman J., Fergusson D., Grimshaw J.M., Hackam D.G. (2013). Threats to validity in the design and conduct of preclinical efficacy studies: A systematic review of guidelines for in vivo animal experiments. PLoS Med..

[96] Liu S., Cheloha R.W., Watanabe T., Gardella T.J., Gellman S.H. (2018). Receptor selectivity from minimal backbone modification of a polypeptide agonist. Proc. Natl. Acad. Sci. USA.

[97] Amrhein F., Lippe J., Mazik M. (2016). Carbohydrate receptors combining both a macrocyclic building block and flexible side arms as recognition units: Binding properties of compounds with CH_2_OH groups as side arms. Org. Biomol. Chem..

[98] Fournie-Zaluski M.C., Gacel G., Maigret B., Premilat S., Roques B.P. (1981). Structural requirements for specific recognition of mu or delta opiate receptors. Mol. Pharmacol..

[99] Zeiss C.J., Allore H.G., Beck A.P. (2017). Established patterns of animal study design undermine translation of disease-modifying therapies for Parkinson’s disease. PLoS ONE.

[100] Santoro M., Fadda P., Klephan K.J., Hull C., Teismann P., Platt B., Riedel G. (2023). Neurochemical, histological, and behavioral profiling of the acute, sub-acute, and chronic MPTP mouse model of Parkinson’s disease. J. Neurochem..

[101] Pagan F.L., Hebron M.L., Wilmarth B., Torres-Yaghi Y., Lawler A., Mundel E.E., Yusuf N., Starr N.J., Arellano J., Howard H.H. (2019). Pharmacokinetics and pharmacodynamics of a single dose Nilotinib in individuals with Parkinson’s disease. Pharmacol. Res. Perspect..

[102] Stone T.W. (2020). Does kynurenic acid act on nicotinic receptors? An assessment of the evidence. J. Neurochem..

[103] Leeson P.D., Baker R., Carling R.W., Curtis N.R., Moore K.W., Williams B.J., Foster A.C., Donald A.E., Kemp J.A., Marshall G.R. (1991). Kynurenic acid derivatives. Structure-activity relationships for excitatory amino acid antagonism and identification of potent and selective antagonists at the glycine site on the N-methyl-D-aspartate receptor. J. Med. Chem..

[104] Zhen D., Liu J., Zhang X.D., Song Z. (2022). Kynurenic Acid Acts as a Signaling Molecule Regulating Energy Expenditure and Is Closely Associated With Metabolic Diseases. Front. Endocrinol..

[105] Foster A.C., Kemp J.A., Leeson P.D., Grimwood S., Donald A.E., Marshall G.R., Priestley T., Smith J.D., Carling R.W. (1992). Kynurenic acid analogues with improved affinity and selectivity for the glycine site on the N-methyl-D-aspartate receptor from rat brain. Mol. Pharmacol..

[106] Stone T.W. (2000). Development and therapeutic potential of kynurenic acid and kynurenine derivatives for neuroprotection. Trends Pharmacol. Sci..

[107] Wu Y., Xie Y., Feng Y., Xu Z., Ban S., Song H. (2023). Diversity-Oriented Biosynthesis Yields l-Kynurenine Derivative-Based Neurological Drug Candidate Collection. ACS Synth. Biol..

[108] Molnár K., Lőrinczi B., Fazakas C., Szatmári I., Fülöp F., Kmetykó N., Berkecz R., Ilisz I., Krizbai I.A., Wilhelm I. (2021). SZR-104, a Novel Kynurenic Acid Analogue with High Permeability through the Blood-Brain Barrier. Pharmaceutics.

[109] Deora G.S., Kantham S., Chan S., Dighe S.N., Veliyath S.K., McColl G., Parat M.O., McGeary R.P., Ross B.P. (2017). Multifunctional Analogs of Kynurenic Acid for the Treatment of Alzheimer’s Disease: Synthesis, Pharmacology, and Molecular Modeling Studies. ACS Chem. Neurosci..

[110] Mándi Y., Endrész V., Mosolygó T., Burián K., Lantos I., Fülöp F., Szatmári I., Lőrinczi B., Balog A., Vécsei L. (2019). The Opposite Effects of Kynurenic Acid and Different Kynurenic Acid Analogs on Tumor Necrosis Factor-α (TNF-α) Production and Tumor Necrosis Factor-Stimulated Gene-6 (TSG-6) Expression. Front. Immunol..

[111] Kovács V., Remzső G., Körmöczi T., Berkecz R., Tóth-Szűki V., Pénzes A., Vécsei L., Domoki F. (2021). The Kynurenic Acid Analog SZR72 Enhances Neuronal Activity after Asphyxia but Is Not Neuroprotective in a Translational Model of Neonatal Hypoxic Ischemic Encephalopathy. Int. J. Mol. Sci..

[112] Nasrallah H.A., Earley W., Cutler A.J., Wang Y., Lu K., Laszlovszky I., Németh G., Durgam S. (2017). The safety and tolerability of cariprazine in long-term treatment of schizophrenia: A post hoc pooled analysis. BMC Psychiatry.

[113] Katzenschlager R., Poewe W., Rascol O., Trenkwalder C., Deuschl G., Chaudhuri K.R., Henriksen T., van Laar T., Lockhart D., Staines H. (2021). Long-term safety and efficacy of apomorphine infusion in Parkinson’s disease patients with persistent motor fluctuations: Results of the open-label phase of the TOLEDO study. Park. Relat. Disord..

[114] Lauriello J., Claxton A., Du Y., Weiden P.J. (2020). Beyond 52-Week Long-Term Safety: Long-Term Outcomes of Aripiprazole Lauroxil for Patients With Schizophrenia Continuing in an Extension Study. J. Clin. Psychiatry.

[115] Ye S., Han Y., Wei Z., Li J. (2023). Binding Affinity and Mechanisms of Potential Antidepressants Targeting Human NMDA Receptors. Molecules.

[116] Bacilieri M., Varano F., Deflorian F., Marini M., Catarzi D., Colotta V., Filacchioni G., Galli A., Costagli C., Kaseda C. (2007). Tandem 3D-QSARs approach as a valuable tool to predict binding affinity data: Design of new Gly/NMDA receptor antagonists as a key study. J. Chem. Inf. Model..

[117] Chou C.K., Liu Y.L., Chen Y.I., Huang P.J., Tsou P.H., Chen C.T., Lee H.H., Wang Y.N., Hsu J.L., Lee J.F. (2020). Digital Receptor Occupancy Assay in Quantifying On- and Off-Target Binding Affinities of Therapeutic Antibodies. ACS Sens..

[118] Batista V.S., Gonçalves A.M., Nascimento-Júnior N.M. (2022). Pharmacophore Mapping Combined with dbCICA Reveal New Structural Features for the Development of Novel Ligands Targeting α4β2 and α7 Nicotinic Acetylcholine Receptors. Molecules.

[119] Lee I.T., Chen S., Schetz J.A. (2008). An unambiguous assay for the cloned human sigma1 receptor reveals high affinity interactions with dopamine D4 receptor selective compounds and a distinct structure-affinity relationship for butyrophenones. Eur. J. Pharmacol..

[120] Morgese M.G., Bove M., Di Cesare Mannelli L., Schiavone S., Colia A.L., Dimonte S., Mhillaj E., Sikora V., Tucci P., Ghelardini C. (2021). Precision Medicine in Alzheimer’s Disease: Investigating Comorbid Common Biological Substrates in the Rat Model of Amyloid Beta-Induced Toxicity. Front. Pharmacol..

[121] Chen F., Li Y., Ye G., Zhou L., Bian X., Liu J. (2021). Development and Validation of a Prognostic Model for Cognitive Impairment in Parkinson’s Disease With REM Sleep Behavior Disorder. Front. Aging Neurosci..

[122] Fan Y., Han J., Zhao L., Wu C., Wu P., Huang Z., Hao X., Ji Y., Chen D., Zhu M. (2021). Experimental Models of Cognitive Impairment for Use in Parkinson’s Disease Research: The Distance Between Reality and Ideal. Front. Aging Neurosci..

[123] Taylor T.N., Greene J.G., Miller G.W. (2010). Behavioral phenotyping of mouse models of Parkinson’s disease. Behav. Brain Res..

[124] Chaira T., Subramani C., Barman T.K. (2023). ADME, Pharmacokinetic Scaling, Pharmacodynamic and Prediction of Human Dose and Regimen of Novel Antiviral Drugs. Pharmaceutics.

[125] Giacomini K.M. (2020). Novel Technologies Enable Mechanistic Understanding and Modeling of Drug Exposure and Response. Clin. Pharmacol. Ther..

[126] Neumaier F., Zlatopolskiy B.D., Neumaier B. (2021). Drug Penetration into the Central Nervous System: Pharmacokinetic Concepts and In Vitro Model Systems. Pharmaceutics.

[127] Obach R.S., Baxter J.G., Liston T.E., Silber B.M., Jones B.C., MacIntyre F., Rance D.J., Wastall P. (1997). The prediction of human pharmacokinetic parameters from preclinical and in vitro metabolism data. J. Pharmacol. Exp. Ther..

[128] Tanaka M. (2024). Beyond the boundaries: Transitioning from categorical to dimensional paradigms in mental health diagnostics. Adv. Clin. Exp. Med..

[129] Nunes Y.C., Mendes N.M., Pereira de Lima E., Chehadi A.C., Lamas C.B., Haber J.F., dos Santos Bueno M., Araújo A.C., Catharin V.C.S., Detregiachi C.R.P. (2024). Curcumin: A golden approach to healthy aging: A systematic review of the evidence. Nutrients.

[130] Chan A., Pirmohamed M., Comabella M. (2011). Pharmacogenomics in neurology: Current state and future steps. Ann. Neurol..

[131] Radonjić N.V., Hess J.L., Rovira P., Andreassen O., Buitelaar J.K., Ching C.R.K., Franke B., Hoogman M., Jahanshad N., McDonald C. (2021). Structural brain imaging studies offer clues about the effects of the shared genetic etiology among neuropsychiatric disorders. Mol. Psychiatry.

[132] Yao X., Glessner J.T., Li J., Qi X., Hou X., Zhu C., Li X., March M.E., Yang L., Mentch F.D. (2021). Integrative analysis of genome-wide association studies identifies novel loci associated with neuropsychiatric disorders. Transl. Psychiatry.

[133] Schrode N., Ho S.M., Yamamuro K., Dobbyn A., Huckins L., Matos M.R., Cheng E., Deans P.J.M., Flaherty E., Barretto N. (2019). Synergistic effects of common schizophrenia risk variants. Nat. Genet..

[134] Xiong S., Li Z., Liu Y., Wang Q., Luo J., Chen X., Xie Z., Zhang Y., Zhang H., Chen T. (2020). Brain-targeted delivery shuttled by black phosphorus nanostructure to treat Parkinson’s disease. Biomaterials.

[135] Lin D., Li M., Gao Y., Yin L., Guan Y. (2022). Brain-targeted gene delivery of ZnO quantum dots nanoplatform for the treatment of Parkinson disease. Chem. Eng. J..

[136] Sela M., Poley M., Mora-Raimundo P., Kagan S., Avital A., Kaduri M., Chen G., Adir O., Rozencweig A., Weiss Y. (2023). Brain-targeted liposomes loaded with monoclonal antibodies reduce alpha-synuclein aggregation and improve behavioral symptoms in Parkinson’s disease. Adv. Mater..

[137] Sim T.M., Tarini D., Dheen S.T., Bay B.H., Srinivasan D.K. (2020). Nanoparticle-based technology approaches to the management of neurological disorders. Int. J. Mol. Sci..

[138] Hu Y., Hammarlund-Udenaes M. (2020). Perspectives on nanodelivery to the brain: Prerequisites for successful brain treatment. Mol. Pharm..

[139] Tukker A.M., de Groot M.W., Wijnolts F.M., Kasteel E.E., Hondebrink L., Westerink R. (2016). Is the time right for in vitro neurotoxicity testing using human iPSC-derived neurons?. ALTEX-Altern. Anim. Exp..

[140] Sirenko O., Parham F., Dea S., Sodhi N., Biesmans S., Mora-Castilla S., Ryan K., Behl M., Chandy G., Crittenden C. (2019). Functional and mechanistic neurotoxicity profiling using human iPSC-derived neural 3D cultures. Toxicol. Sci..

[141] Tukker A.M., Wijnolts F.M., de Groot A., Westerink R.H. (2018). Human iPSC-derived neuronal models for in vitro neurotoxicity assessment. Neurotoxicology.

[142] Woodruff G., Phillips N., Carromeu C., Guicherit O., White A., Johnson M., Zanella F., Anson B., Lovenberg T., Bonaventure P. (2020). Screening for modulators of neural network activity in 3D human iPSC-derived cortical spheroids. PLoS ONE.

[143] De Paola M., Pischiutta F., Comolli D., Mariani A., Kelk J., Lisi I., Cerovic M., Fumagalli S., Forloni G., Zanier E.R. (2023). Neural cortical organoids from self-assembling human iPSC as a model to investigate neurotoxicity in brain ischemia. J. Cereb. Blood Flow. Metab..

[144] Tanaka M. (2025). From Serendipity to Precision: Integrating AI, Multi-Omics, and Human-Specific Models for Personalized Neuropsychiatric Care. Biomedicines.

